# Genetics Selection Evolution: news for the period January 2009 - May 2010 and editorial announcements

**DOI:** 10.1186/1297-9686-42-15

**Published:** 2010-05-28

**Authors:** Helene Hayes, Didier Boichard

**Affiliations:** 1 INRA, UMR 1313 Génétique Animale et Biologie Intégrative, F78350 Jouy-en-Josas, France

## 

It is now nearly a year and a half since the journal *Genetics Selection Evolution *(GSE) is published as an open access journal and it is time for an update on its activity and to share some changes on the editorial board.

Comparing statistics between 2008 and 2009 provides some initial indication on the consequences of the "Open Access" conversion.

The total number of manuscripts submitted in 2008/2009 was similar i.e. 98/93. Except for manuscripts originating from China (22 in 2008/12 in 2009), there was little variation between 2008 and 2009: origin and number of manuscripts were quite stable with a large range of origins concentrating over Norway (8 in 2008/10 in 2009), USA (8/9), the Netherlands(7/10), France (7/8), Spain (6/5), Australia (3/6), UK (3/4), Germany (2/4) and Italy (1/4). Thus, in 2009 introduction of an author pay charge (APC) had little effect.

The total number of manuscripts published was higher in 2009 (54) than in 2008 (40), which is partly due to the success of the call for sponsored papers in spring 2008 to promote new Open Access GSE *i.e*. eight sponsored papers were published. The increase in number of published papers is made possible by the fact that with the Open Access online model, there is no restriction in page number as before with the paper version.

Over 2008 and 2009, the number of submissions per field was as follows: methodological and quantitative genetics (25 in 2008/37 in 2009), applied and experimental genetics (25/29), population genetics and genetic diversity (24/12), molecular genetics (16/7) and others (8/8). Thus, about three quarters of the submitted manuscripts concerned in equal proportions methodological, applied and population genetics in 2008 while in 2009, we observed a clear increase in manuscripts dealing with methodological and quantitative genetics and a clear decrease in manuscripts on genetic diversity.

Clearly, GSE is established as a journal specialized on the quantitative and applied aspects of genetics and selection in farm animals.

GSE works with many peer reviewers all experts in their own field. Thorough peer reviewing is essential to guaranty the quality and the relevance of the papers published in GSE. We are well aware that reviewing manuscripts is a heavy and time-consuming task and we are grateful to all the referees for supporting GSE in this way.

We have decided to go further and to offer referees, sending back their review on time, a 20% discount on author pay charge if they publish an article in Genetics Selection Evolution. This offer will hold for a year after submission of the review.

Another change is the possibility for referees to upload an attached file with their evaluation report, which is useful in cases where special symbols and mathematical equations are necessary.

Over 2009 and 2010, several changes have taken place on the editorial board:

- (1) three associate editors **André Eggen **(Jouy-en-Josas, France), **Catherine Montchamp-Moreau **(Gif-sur-Yvette, France) and **Miguel Perez-Enciso **(Bellatera, Spain) have retired from the editorial board and we thank them warmly for their active collaboration with the journal during many years

- (2) seven new associate editors have joined us bringing their expertise in their respective fields of interest:

▪ **Paolo Ajmone-Marsan **(Piacenza, Italy): animal molecular genetics applied to the investigation of genetic diversity and to the identification of genes and QTL controlling economic traits

▪ **Armando Caballero **(Vigo, Spain): strategies for controlling rates of inbreeding in selection and conservation programs, genetic variability in natural populations, estimation of mutational parameters in quantitative traits and their evolutionary consequences

▪ **Mario Calus **(Lelystad, The Netherlands): statistical and quantitative genetics, QTL detection, genomic selection, genomic breeding value estimation

▪ **Christa Kühn **(Dummerstorf, Germany): molecular bovine genetics, QTL identification, association of polymorphism with breeding traits

▪ **Ricardo Pong-Wong **(Edinburgh, UK): statistical and quantitative genetics, marker assisted selection, inbreeding

▪ **Alain Vignal **(Castanet-Tolosan, France): avian molecular genetics, QTL detection, genome mapping

▪ **Lusheng Huang **(Jiangxi, P.R. China): pig genetics and genomics, QTL analysis for production traits.

Last but not least, a new executive co-editor for GSE!

After four and a half years as scientific and now executive editor of GSE, **Philippe Baret **will step down from this position to devote more time to his research and academic activities. Over the period, he has been actively involved in all the editorial decisions of GSE and especially in the successful adventure of moving GSE to Open Access. It has been a great pleasure to work with Philippe and we thank him for contributing his time and effort to the good management of GSE and for bringing GSE to where it is today.

We are pleased to announce that **Jack Dekkers **from Iowa State University (USA) has agreed to join the editorial board of GSE as our new executive editor. **Jack Dekkers **is professor of animal breeding and genetics and is internationally recognized for his work on quantitative genetics and animal breeding applied to dairy cattle, swine and poultry.

In his own words: "I am excited to join the GSE team because I agree with the philosophy and focus of GSE and hope I can do my part to ensure its continued success".

**Jack Dekkers **will take up his position with GSE from mid-May 2010. We welcome him to the journal and look forward to working with him.

